# Integrating Protein Language Models with Multimodal Embeddings to Accelerate Function Prediction of Uncharacterized Proteins

**DOI:** 10.3390/ijms27093891

**Published:** 2026-04-27

**Authors:** Ruyang Cheng, Tianyu Liu, Chentao Liao, Xiaomin Wu, Lingyun Zhu, Shaowei Zhang

**Affiliations:** College of Science, National University of Defense Technology, Changsha 410073, Chinawuxiaomin@nudt.edu.cn (X.W.)

**Keywords:** protein structure–function, protein language models, multimodal, protein function prediction, chemical reaction space

## Abstract

Accurate prediction of protein function is fundamental to progress in biotechnology and biomedicine, yet progress remains severely hampered by the widening chasm between exponentially growing genomic data and the limited capacity for functional annotation. High-throughput sequencing and metagenomics have driven an explosion in sequence data that far outstrips experimental characterization. UniProt now contains over 203 million protein entries, of which only ~2% have been experimentally validated. This widening “sequence–function gap” exceeds the reach of traditional homology-based tools such as BLAST (v2.17.0) and HMMER (v3.2), which are inherently constrained by sequence identity thresholds. The emergence of Protein Language Models (PLMs), including ESM and ProtTrans, has introduced a transformative paradigm, thereby shifting functional inference from similarity-based retrieval to geometric reasoning within learned semantic spaces. Nevertheless, current approaches remain largely confined to unimodal or narrowly bimodal frameworks, failing to capture the inherently multidimensional determinants of enzymatic function, including active-site geometry, chemical reaction logic, and literature-embedded semantic context. This review systematically adopts a multimodal global-fusion perspective, elucidating how three-dimensional geometric features, chemical reaction semantics, and textual knowledge graphs are synergistically integrated around PLMs as a core backbone. We delineate complementary mechanisms and integration strategies that together enable fine-grained protein function annotation beyond the performance ceiling of single-sequence methods. Furthermore, we survey the translational potential of such frameworks from computational prediction to real biological applications, and critically examine persistent bottlenecks including activity cliffs, transition-state inference, and conformational dynamics. We identify the integration of physics-informed machine learning with dynamics-aware architectures as a pivotal direction toward a causal, mechanism-level understanding of protein function.

## 1. Introduction

Proteins serve as the fundamental effectors of biological systems, exhibiting diverse and complex functions ranging from catalyzing reactions and regulating signaling pathways to maintaining cellular architecture. Consequently, protein functional annotation represents a pivotal link bridging genomic information with biological phenotypes, impacting diverse fields from fundamental research to synthetic biology. Specifically in the field of enzyme mining, such annotation facilitates the discovery of biocatalysts with novel activities. Notably, microbial communities possess tremendous enzymatic potential, and through function-driven enzyme discovery, enzymes displaying specific environmental activity (or in vivo activity) can be identified from these natural communities [1]. Complementing these discovery efforts, enzyme engineering aims to further optimize critical properties such as stability and catalytic efficiency by modifying amino acid sequences, or even seeks to develop de novo catalytic activities not found in nature [2,3]. Crucially, accurate protein functional annotation is indispensable for guiding such directed evolution and engineering efforts [4]. Extending to the realm of drug design, functional annotation aids in identifying therapeutic targets and predicting protein-ligand interactions, thereby accelerating the drug development process. Moreover, a deep understanding of protein sequence and structure is fundamental to elucidating protein–protein interactions (PPIs), which play a central role in numerous biological processes and disease mechanisms [5]. Consequently, developing advanced computational strategies beyond traditional homology-based approaches is imperative to annotate the vast expanse of uncharacterized proteins, thereby unlocking new frontiers in synthetic biology and precision medicine.

**Figure 1:**
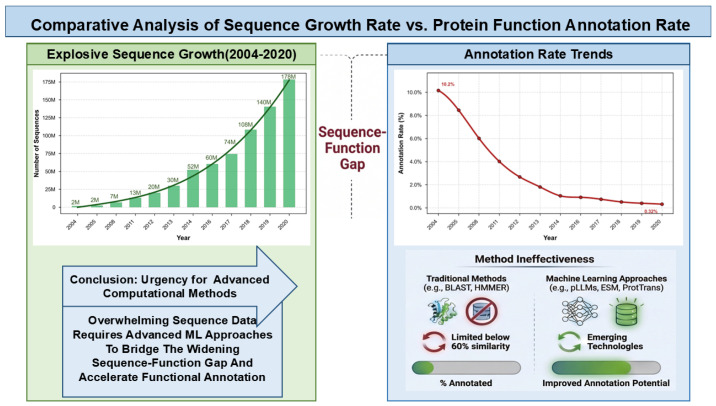
Comparative analysis of sequence growth rate versus protein function annotation rate reveals an expanding sequence–function gap. (**Left**) Exponential growth of protein sequences in UniProt (2004–2020), driven by large-scale meta-genomic sequencing projects. Key milestones: 1.5 million sequences in 2004, 30 million in 2013 (Illumina sequencing revolution), and 178 million in 2020. (**Right**) Declining trend of experimentally validated functional an-notations. The annotation rate has dropped from >10% (2004) to <1% (2020). Data were sourced from relevant literature and UniProt release notes (ftp://ftp.ebi.ac.uk/pub/databases/uniprot/previous_major_releases/) accessed on 20 January 2020 [8,9]. The Swiss-Prot annotation rate is presented as a conservative estimate of experimentally validated sequences.

In recent years, the rapid advancement of high-throughput sequencing technologies, particularly the widespread application of metagenomics, has generated massive amounts of protein sequence data [6]. However, a substantial fraction of these sequences often termed “dark matter” proteins remains devoid of reliable functional annotation. Currently, the UniProt database houses exceeding 203 million entries, of which merely ~2% have been experimentally validated. A striking example is found in Glycoside Hydrolases (GHs), the largest superfamily in the CAZy database essential for carbohydrate turnover. While over 990,000 GH domains across 171 families have been mined from sequence data, only a minute fraction (~7200 modules, or 0.73%) is supported by experimental evidence [7]. As illustrated in Figure 1, this immense disparity where exponential sequence growth far outpaces experimental validation results in a widening ‘Sequence–Function Gap’ that underscores the urgent need for advanced computational strategies.

Traditional functional annotation relies on sequence homology alignment tools like BLAST (v2.17.0) and HMMER (v3.2), grounded in the assumption of a strong correlation between sequence identity and functional similarity. BLAST infers function by aligning target sequences against annotated databases to identify high-similarity hits [10], while HMMER utilizes profile hidden Markov models (pHMMs) to detect conserved patterns, offering superior sensitivity for identifying remote homologs compared to BLAST [11]. Despite their widespread application, their reliability is strictly constrained by sequence identity thresholds. Reliable functional transfer typically requires sequence similarity above 60% [10]. However, biological reality is more complex: protein structure is evolutionarily more conserved than sequence [12], and common ancestry does not strictly dictate identical function. Consequently, traditional alignment methods often fail to accurately characterize proteins with low sequence identity to known enzymes. This limitation has exacerbated the massive ‘Sequence–Function Gap’, impeding our comprehensive understanding of the protein universe and thereby highlighting the critical need for methods that go beyond simple sequence alignment.

**Figure 2:**
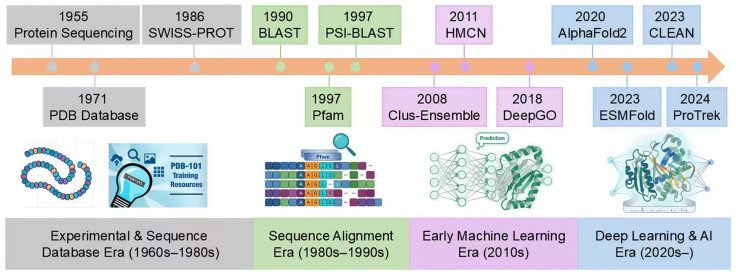
History of protein function prediction methods [15,16,17,18,19,20]. From the 1960s to the present, computational approaches have progressed through four major phases: experimental work and sequence database construction, sequence alignment and domain recognition, early machine learning, and the current era of deep learning and artificial intelligence. Key methods or databases for each phase are annotated on the timeline. The emergence of protein language models represents a major paradigm shift: functional inference has moved from reliance on homology-based alignment to geometric reasoning in high-dimensional semantic spaces, enabling annotation of remote homologs and “orphan” proteins. Subsequently, multimodal (the integration of protein sequences, structural information, and natural language text) integration has further unified sequence, structure, biochemical reactions, and textual knowledge into joint representation learning, substantially improving predictive accuracy and generalization.

To surmount the limitations of traditional alignment methods, Machine Learning (ML) and Deep Learning (DL) models were initially introduced into protein function prediction. While achieving progress in specific tasks, these approaches often suffer from limited feature extraction capabilities relying heavily on hand-crafted or local features and lack robust generalization [13,14]. Across this evolutionary trajectory (Figure 2), computational approaches have iterated from experimental database construction to sequence alignment and then to machine learning. However, the true paradigm shift is marked by the advent of protein language models, which transform functional inference from homology-threshold-based “similarity retrieval” to “geometric reasoning” in high-dimensional semantic spaces.

Recently, inspired by breakthroughs in Natural Language Processing (NLP), Protein Language Models (PLMs), such as ESM [21] and ProtTrans [22], have emerged as a transformative paradigm. By leveraging self-supervised pre-training on massive unannotated corpora, these models generate embeddings (a method for converting text (e.g., words or sentences) into numerical vectors) that effectively capture deep contextual semantics and long-range dependencies within protein sequences. This potent representation capability has rapidly catalyzed breakthroughs in downstream tasks. For instance, Yu et al. introduced CLEAN [23], a model that innovatively combines ESM embeddings with contrastive learning, significantly enhancing annotation accuracy for rare enzymes. Notably, pushing the boundaries of multimodal integration, ProTrek [24] introduces a trimodal language model that unifies protein sequence, 3D structure, and natural language descriptions through contrastive learning. This architecture outperforms established tools like Foldseek and MMseqs2 in both speed and accuracy for functional retrieval across billion-scale repositories.

Collectively, these advances underscore that the efficacy of downstream prediction hinges on the quality of the underlying sequence representation. This necessitates a fundamental examination of how PLMs transform raw biological sequences into computable features, laying the groundwork for more complex multimodal integration.

This review therefore addresses three interconnected challenges: (1) how PLMs construct biologically meaningful representations from raw sequences; (2) whether the systematic integration of orthogonal modalities—structural geometry, chemical reaction logic, and textual semantics—anchored on PLMs as the core representational backbone—can collectively overcome the performance ceiling imposed by unimodal sequence-based frameworks; and (3) what computational and physical bottlenecks must be resolved to advance from statistical correlation toward mechanism-level understanding of protein function.

Here, we elucidate how integrating Protein Language Models (PLMs) with multimodal embeddings accelerates the functional inference of uncharacterized proteins. We first delineate the computational paradigms enabling PLMs to reconstruct high-dimensional semantic manifolds, explicitly linking the implicit encoding of evolutionary histories to the geometric clustering of functional phenotypes. We then systematically explore how integrating orthogonal modalities accelerates functional inference: capturing catalytic pocket topology via geometric embeddings, bridging sequence space with chemical transformation logic, and empowering zero-shot reasoning through textual knowledge integration. Furthermore, we highlight the translation of these frameworks from in silico benchmarking to tangible wet-lab validation, successfully guiding the functional elucidation of uncharacterized proteins and correcting historical mis-annotations. Finally, we discuss critical bottlenecks such as “activity cliffs” (structurally similar molecules exhibit markedly different activities) and time-scale gaps, proposing physics-informed and dynamics-aware learning as the future frontier for decoding the protein universe.

## 2. Protein Language Models: Constructing High-Dimensional Semantic Spaces

Protein sequences serve as the genetic blueprint dictating structure and function, encapsulating a wealth of biological information. However, this information is intrinsically latent within the linear arrangement of amino acids, often eluding direct interpretation by conventional methodologies. Consequently, in the context of PLM-based enzyme functional annotation, the central challenge lies in effectively transmuting biological sequences into computable, high-dimensional digital representations, thereby enabling the efficient decoding of implicit evolutionary patterns and physicochemical properties.

Initial efforts to operationalize these sequences utilized One-hot encoding, which treats amino acids as discrete, orthogonal variables—a method exemplified by early models like DeepEC [25]. However, given the inability of One-hot encoding to capture physicochemical correlations between residues, the Position-Specific Scoring Matrix (PSSM), derived from Multiple Sequence Alignment (MSA), emerged as a superior alternative. By incorporating evolutionary conservation profiles, PSSM has long been regarded as the “gold standard” for feature extraction. However, this reliance on MSA proved to be a double-edged sword. The computational cost of MSA is prohibitive for today’s exponentially expanding databases, and critically, it fails for “orphan proteins” that lack homologous counterparts. In recent years, the rise of Protein Language Models (PLMs) has bridged this gap. By transferring deep learning architectures from NLP, PLMs autonomously learn the “grammar” of protein sequences through self-supervised training, enabling the efficient extraction of latent high-dimensional biological features without reliance on MSA.

### 2.1. Language Models as Semantic Feature Extractors for Proteins

Protein Language Models (PLMs) aim to reconstruct the feature space of biological sequences by mapping discrete amino acid sequences into continuous high-dimensional vectors (embeddings), thereby constructing a computable protein semantic space (a high-dimensional abstract space formed by embedding vectors) [26]. Their technical architecture is profoundly inspired by classic paradigms in Natural Language Processing (NLP) and specifically adapted to the characteristics of biological sequences. Among these, Masked Language Models (MLMs), represented by the BERT [27] and ESM [17,21] series, adopt a bidirectional training strategy. By randomly masking a fraction of residues (typically 15%) in the input sequence and reconstructing them, these models are forced to deeply parse the dependency of each amino acid on its global context, accurately capturing complex long-range interactions within the protein [27]. Conversely, Autoregressive Language Models, represented by ProtGPT2, focus on unidirectional generation tasks, predicting the next amino acid based on the preceding context [28]. This paradigm not only emulates the generative process of sequences but also strengthens the capture of implicit causal logic. Through extensive pre-training on massive corpora, the co-evolutionary patterns embedded in homologous sequences enable these models to implicitly learn statistical dependencies that correspond to spatial proximity in three-dimensional structures. As demonstrated by Lin et al. [17], given that ESM-2 is trained exclusively on sequences, any structural information emerging within the model necessarily reflects the representation of co-evolutionary patterns encoded in the sequence itself—a conclusion directly supported by the striking correspondence between the model’s self-attention maps and residue–residue contact maps, a correspondence that strengthens systematically with increasing model scale [21]. Consequently, the hidden layers implicitly encode evolutionary history, structural constraint, and latent functional information into high-dimensional “semantic vectors”, achieving a potent compression from discrete symbols to a continuous numerical space [21].

### 2.2. Emergence of Protein Structural Encoding in High-Dimensional Semantic Spaces

Remarkably, although trained exclusively at the sequence level, a capacity for encoding protein tertiary structure has spontaneously emerged within the high-dimensional semantic spaces of PLMs. At its core, this “structural emergence” derives from the physical constraints imposed by natural evolution: co-evolutionary signals between distant residues are intrinsically maintained to preserve spatial contacts and structural stability within the three-dimensional fold [21,29]. By capturing these subtle statistical regularities through deep attention mechanisms, PLMs implicitly encode the evolutionary fingerprints of structural constraints, rather than explicitly learning the underlying physical laws of protein folding [17,21,30]. Consequently, high-accuracy protein structure prediction requires explicit architectural components—such as geometric modeling modules or equivariant folding frameworks—to translate these statistical representations into three-dimensional coordinates, as exemplified by ESMFold [17] and HelixFold-Single [31], both of which integrate PLM-derived representations with dedicated geometric modeling components to achieve accurate structure prediction. In summary, the high-dimensional vectors generated by PLMs serve not only as mathematical representations of sequences but also as bridges connecting one-dimensional linear sequences to three-dimensional topological structures. They provide rich sequence-derived representations that, when combined with appropriate geometric modeling, enable efficient and accurate structure prediction.

### 2.3. Implicit Compression of Evolutionary Information

In contrast to traditional PSSMs, which rely on the explicit construction of Multiple Sequence Alignments (MSAs) to extract co-evolutionary signals, Protein Language Models (PLMs) introduce a highly efficient mechanism of “implicit evolutionary modeling.” According to Rives et al. [21], through self-supervised pre-training on billions of protein sequences, PLMs extract latent evolutionary constraints such as amino acid conservation and co-variation from massive datasets, transforming them into internal, computable mathematical features. The biological essence of this mechanism lies in the fact that when the model recovers masked residues based on context, it is effectively performing evolutionary inference governed by statistical laws.

Notably, although BERT-style models process only single sequences during training without exposure to MSAs, studies have revealed a striking alignment between their deep Self-Attention Maps and the Residue Contact Maps derived from MSAs. This phenomenon confirms that the model has successfully implicitly “compressed” co-evolutionary signals traditionally accessible only through homologous sequence alignment into the weights of the neural network [30]. This “alignment-free” paradigm not only eliminates the prohibitive computational cost of MSA construction but also circumvents the bottleneck of missing homologous information for “orphan proteins”, significantly enhancing the universality and efficiency of feature extraction across the entire proteome.

### 2.4. Clustering of Functional Semantics

The effective encoding of structural and evolutionary information culminates in the emergence of distinct functional clusters within the high-dimensional semantic space. Here, the fundamental biological principle that “structure dictates function” is translated into a mathematical reality: functionally analogous proteins are represented by vectors that are geometrically proximal. Evidence from the CLEAN model demonstrates that enzymes sharing identical EC numbers maintain compact clustering in the ESM-2 latent space, remarkably persisting even when sequence identity drops below the “twilight zone” threshold of 25% [23]. This suggests that function-constrained evolutionary pressure compels diverse sequence motifs to converge in the deep semantic manifold [32]. Consequently, proteins with distinct functions spontaneously segregate into sharply defined clusters. This phenomenon lays a solid mathematical cornerstone for annotation: assigning function to an unknown protein becomes a geometric task of quantifying spatial proximity via metrics like cosine similarity to annotated clusters, or defining decision hyperplanes that separate functional classes [33].

**Figure 3:**
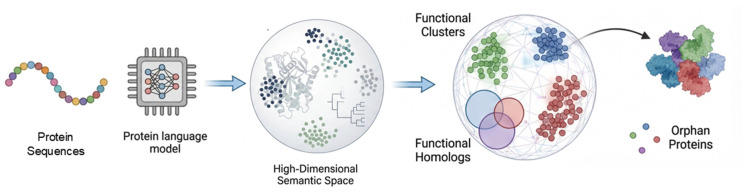
High-Dimensional Semantic Spaces from Protein Language Models. Discrete protein sequences are encoded into high-dimensional embeddings by protein language models, during which three-dimensional structural encoding spontaneously emerges and evolutionary information is implicitly compressed through cross-species statistical patterns, jointly constituting the foundation of the high-dimensional semantic space. Functionally similar proteins form compact clusters (functional homologs) in the semantic space, maintaining geometric proximity even with extremely low sequence identity; orphan proteins (lacking homologous sequences) can also be effectively represented in this space, enabling functional annotation without multiple sequence alignment.

As summarized in Figure 3, this framework integrates the spontaneous emergence of structural encoding and the implicit compression of evolutionary information to construct a semantic space where functional annotation becomes a solvable geometric problem.

## 3. Achieving Fine-Grained Protein Function Annotation via Multimodal Feature Embeddings

Despite the robust sequence representations offered by protein language models (PLMs), Capela et al. argue that feature extraction represents only the foundational step [34]. While their study confirms that PLMs vastly surpass One-hot encoding in generalizability, it also exposes a critical limitation: simple linear classifiers are insufficient to bridge the semantic gap mapping raw sequence features to complex functional labels. This is particularly evident in high-homology tasks, where unoptimized PLMs downstream models frequently hit a performance plateau, yielding results merely comparable to traditional BLASTp alignment.

**Figure 4:**
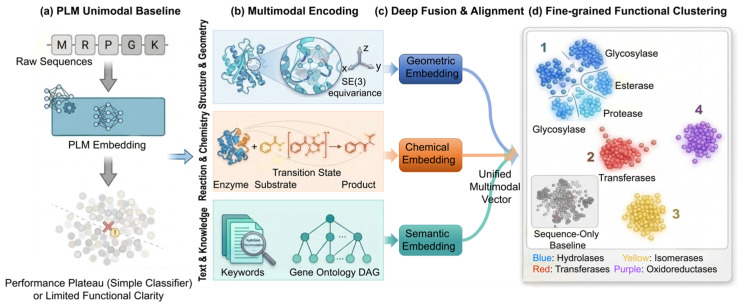
Multi-modal Feature Embedding for Refined Protein Function Annotation. (**a**) The PLM unimodal baseline relies solely on raw sequence embeddings, which encounter performance plateaus when using simple classifiers and exhibit limited functional clarity (overlapping clusters). (**b**) Multimodal information encoding: SE(3)-equivariant networks extract 3D geometric features, transition-state mechanisms encode enzyme–substrate reaction chemistry, and keywords with Gene Ontology DAGs extract textual semantic features. (**c**) Deep fusion and alignment: cross-attention and space alignment project three-modality embeddings into a unified multimodal vector. (**d**) Fine-grained functional clustering: proteins are distinctly separated by functional families in the unified embedding space (blue: Hydrolases and subtypes; red: Transferases; yellow: Isomerases; purple: Oxidoreductases), significantly outperforming the sequence-only baseline.

The root of this limitation lies in the complexity of biological function. Enzymatic activity is strictly contingent upon the precise geometric conformation of the active site, the physicochemical milieu of the substrate-binding pocket, and the synergistic interplay with cofactors—multidimensional structural and chemical contexts that are often lost in a purely sequence-based modality. Consequently, to break through the performance ceiling of single-sequence approaches and accelerate the realization of high-precision enzyme annotation, current methodologies must evolve beyond the unimodal framework. This necessitates the integration of complex architectures capable of fusing and decoding multimodal embeddings [35]. As illustrated in Figure 4, this multimodal paradigm integrates 3D geometric features, chemical reaction logic, and textual semantics to achieve fine-grained functional clustering that significantly outperforms sequence-only baselines.

### 3.1. Structural and Geometric Feature Enhancement

Although protein language models (PLMs) capture evolutionary structural constraints and implicitly learn inter-residue contact patterns through pre-training on massive sequence datasets, this statistical inference based on co-evolution remains limited when addressing the intricate mechanisms of enzymatic function. Enzyme substrate specificity and catalytic efficiency are fundamentally dictated by the physicochemical micro-environment of the active site and the precise spatial arrangement of side-chain atoms, rather than merely by the overall topological fold of the backbone.

The advent of AlphaFold2 [16] and ESMFold [17] has triggered an explosive growth in structural data, providing a pivotal opportunity to surmount this bottleneck. Explicit structural inputs introduce physicochemical information inaccessible to sequence motifs alone, including atomic coordinates, electrostatic potential distributions, van der Waals interactions, and the geometry of active cavities. Consequently, residue-level embeddings extracted by PLMs can be significantly augmented by fusing them with geometric information, thereby establishing a serial “sequence–structure” processing architecture that enhances prediction accuracy for both functional sites and global protein properties.

To implement this integration, the prevailing methodology utilizes deep sequence embeddings from PLMs as high-quality node features, which are subsequently processed by Graph Neural Networks (GNNs) to aggregate topological information within spatial neighborhoods. GNNs represent the most intuitive approach for structural encoding, modeling proteins as graphs where nodes represent atoms or residues and edges denote covalent bonds or spatial proximity. Early attempts such as DeepFRI [36] demonstrated the viability of this approach by applying Graph Convolutional Networks (GCNs) to contact maps for Gene Ontology (GO) prediction. Moving beyond simple contacts, GPSFun [37] constructs geometric graphs directly from ESMFold-predicted structures to enhance local micro-environment perception. Building on this foundation, ESM-GearNet [38] further validated the synergy between evolutionary semantics and geometric encoders, proving that injecting ESM-2 representations into a geometry-specialized GearNet yields superior performance. However, to mitigate the issue of global structural redundancy, GraphEC [39] refined this paradigm by introducing an active-site probability map as prior knowledge, thereby forcing the model to shift attention from the global fold to the specific local pockets where catalysis occurs.

Beyond capturing topological connectivity, a critical challenge for high-precision prediction lies in handling the rotational and translational invariance of enzyme active pockets in 3D space. To address this, TransFun [40] adopts an equivariant modeling strategy, combining ESM-1b with an SE(3)-equivariant Graph Neural Network (EGNN, a Semantic Space neural network whose output features transform in the same way as the input 3D structure under rotations or translations, ensuring robustness to spatial transformations) to ensure feature extraction remains consistent regardless of coordinate rotation. Taking a different approach to integration, LM-GVP [41] employs an end-to-end cascading architecture where structure serves as an inductive bias; it utilizes a Geometric Vector Perceptron (GVP) as a structure-aware prediction head to strictly guide the language model’s output. More recently, frontier research has sought to bridge the gap between modalities by encoding continuous 3D structures into discrete sequences. SaProt [42] proposes a “Structure Tokenization” paradigm, utilizing Foldseek to discretize continuous 3D structures into tokens aligned with the sequence. This innovative approach allows the model to process structural information in a language-like manner, achieving a deep semantic alignment between residue sequences and 3D conformations within a unified framework.

### 3.2. Alignment of Enzyme–Substrate and Reaction Chemical Spaces

While embeddings of sequence and structure significantly enhance protein function prediction, the functional essence of an enzyme is defined not only by its physical architecture but also fundamentally by the biochemical reaction it catalyzes. Traditional prediction models often treat enzyme function as a discrete classification label, ignoring the explicit chemical transformation logic between reactants and products. To bridge this semantic deficiency, recent research has pivoted towards integrating “enzyme-reaction” information by transforming chemical reactions into computable high-dimensional vectors and aligning them with protein embeddings. To achieve this cross-modal alignment, architectures such as CLIPZyme [43], ReactZyme [44], and FusionESP [45] adopt a dual-tower encoding strategy, aiming to align the biological space of enzymes with the chemical space of reactions. Specifically, CLIPZyme [43] advances beyond static molecular graphs by grounding its approach in biophysical principles; it utilizes atom-mapping techniques to construct a “pseudo-transition state” graph that explicitly models bond breaking and formation. In contrast, ReactZyme [44] views reactions as dynamic transformation processes without relying on manual atom-mapping rules, instead introducing a cross-attention mechanism between substrate and product graphs to implicitly learn bond transformation features. Distinct from these graph-heavy approaches, FusionESP [45] demonstrates a “pure language model” paradigm, projecting substrate chemical semantics and enzyme evolutionary features into a shared manifold via non-linear projection heads to capture stereochemical compatibility without expensive 3D data.

Moving from general alignment to fine-grained interaction under the supervised learning paradigm, models like VIPER [46] and MEI [47] introduce more complex mechanisms to enhance specificity. VIPER [46] utilizes a Cross-Attention module to force enzyme features to dynamically focus on critical substrate substructures. Crucially, beyond architecture, VIPER innovates at the data level by utilizing the RetroRules database to filter out chemically impossible reactions, ensuring the model learns universal Enzyme–Substrate Reaction Mechanics rather than statistical artifacts. Addressing a different challenge regarding specificity, RC-GNN [48] focuses on the Reaction Center. It innovatively employs graph augmentation to aggregate the local chemical environment around the reaction center, simulating the enzyme’s specific recognition mechanism. Furthermore, by constructing “decoy” negative samples with high chemical similarity, RC-GNN forces the model to master regioselectivity, thereby breaking the bottleneck of distinguishing highly similar metabolic reactions.

Finally, extending the methodology to data-centric strategies, CATNIP [49] showcases a novel approach connecting chemical space and protein sequence space to predict biocatalytic reactions. Diverging from standard PLM fine-tuning routes, this study leverages high-quality datasets from high-throughput experimentation. It combines Sequence Similarity Networks (SSNs) for enzyme features with MORFEUS for substrate chemical space features, feeding them into a Gradient Boosting Model (GBM). This strategy successfully achieves precise, bidirectional prediction of enzyme–substrate compatibility, demonstrating that aligning chemical and biological spaces can be effectively realized through high-quality experimental data even in the absence of complex deep learning architectures.

### 3.3. Deep Integration of Textual Semantics and Knowledge Graphs

Beyond physicochemical attributes, scientific literature and ontologies contain a wealth of textual knowledge that provides a macro-semantic backdrop for understanding protein function. Integrating Natural Language Processing (NLP) techniques with PLMs marks a paradigm shift from simple “label classification” to genuine “semantic comprehension.” Spearheading the alignment of sequence and text, models such as ProtST [50] and ProTrek [24] harness high-dimensional sequence features from PLMs (like ESM) and spatially align them with functional descriptions or biomedical abstracts encoded by PubMedBERT [51]. Specifically, ProtST [50] not only achieves global representation alignment via Contrastive Learning but also innovates with a “multimodal mask prediction” task. By using cross-attention mechanisms to capture fine-grained dependencies—such as associating hydrophobic residues with the term “thermal stability”—it empowers the model with natural language understanding. This multi-granular training enables the model to utilize text prompts for zero-shot prediction of unseen labels and facilitates natural language-based protein retrieval, effectively solving the “long-tail” problem that traditional multi-label classification fails to address. Building upon this, ProTrek [24] further incorporates protein structure as a third modality, establishing a comprehensive “sequence–structure–function” tri-modal contrastive learning framework.

However, purely sequence-based or text-based models often overlook the complex dependencies between functional labels, such as the Directed Acyclic Graph (DAG) structure of Gene Ontology (GO). Addressing this limitation, studies like OntoProtein [52] and KeAP [53] integrate Knowledge Graphs (KGs) comprising proteins, GO terms, and their entity relationships. By introducing Knowledge Reconstruction tasks during pre-training, these methods force the PLM’s latent representations to encode implicit biological logical structures. Advancing this logic further, DeepGO-SE [54] combines ontology axioms with sequence features, modeling function prediction as a semantic entailment problem, thereby mathematically guaranteeing the logical consistency of the predicted results.

Beyond static embeddings, recent paradigm shifts have explored retrieval-augmented and generative approaches. ProtEx [55] and ProtIR [56] demonstrate that utilizing retrieved homologous exemplars and their textual annotations as context can significantly boost performance on low-homology sequences. Simultaneously, aiming for an end-to-end solution, Prot2Text [57] and InstructProtein [58] explore generative frameworks. By combining GNNs to process structural information with Transformers for text generation, these models achieve a leap from merely “predicting label IDs” to “generating coherent functional description texts”, providing human-readable insights into protein function.

### 3.4. Translational Potential: From Computational Benchmarking to Real Biological Discovery

Notably, these multimodal frameworks have transitioned from theoretical benchmarking to tangible wet-lab validation, successfully guiding the functional elucidation of uncharacterized proteins and correcting historical misannotations.

For instance, the DeepFRI model developed by Gligorijević et al. integrates sequence representations from protein language models with structural graph information to successfully predict the functions of several unknown microbial proteins, such as the 4Fe-4S cluster-binding function of an Fe-S cluster hydrogenase (PDB ID: 6FOK) and the DNA-binding and metal ion-binding functions of a zinc finger protein (PDB ID: 1MEY). These predictions were subsequently validated through in vitro enzymatic assays [36]. This demonstrates that such models have become reliable hypothesis generators for mining novel enzyme functions from metagenomic data. In connecting chemical and biological spaces, models such as ReactZyme and CLIPZyme align enzyme sequence embeddings with feature representations of chemical reactions, enabling the intelligent recommendation of potential catalysts for target reactions and opening new avenues for biocatalyst discovery [43,44].Going a step further, by semantically aligning protein sequences with functional descriptions (as exemplified by the ProtST model), researchers can perform zero-shot prediction of functional labels not seen during training and retrieve relevant proteins using natural language queries, significantly enhancing the ability to handle novel proteins and complex functional descriptions [59]. For example, using ProtST to perform zero-shot retrieval and visualization for heme-binding proteins, the top three candidate proteins were all annotated as heme-binding proteins in the Gene Ontology (GO) database. At the finest scale, ESM-GearNet combines the sequence representations from ESM-2 with the geometric encoder of GearNet, utilizing a serial fusion strategy to effectively integrate evolutionary semantics with structural features. Instead of relying on a single modality, this framework leverages joint pre-training on the AlphaFold Database to capture the mutual dependencies between sequence and structure. Comprehensive benchmarking demonstrates that ESM-GearNet significantly outperforms unimodal models, establishing new state-of-the-art performance in protein function prediction tasks, particularly for Enzyme Commission (EC) number and Gene Ontology (GO) term annotations [60]. FusionESP further integrates ESM-2 with MoLFormer, aligning enzyme sequences and substrate chemical spaces through contrastive learning to achieve a prediction accuracy of 94.77% on an independent test set, implicitly capturing stereochemical compatibility without requiring expensive 3D data [45]. In contrast, traditional sequence homology-based retrieval approaches, such as DIA-MOND BLASTp, still exhibit notable limitations in the recognition and functional annotation of certain proteins, particularly those with low sequence similarity or substantial functional divergence [61].

Multimodal models are progressively transitioning from proof-of-concept demonstrations to practical solutions for real biological problems, and have shown the potential to outperform traditional approaches across multiple cutting-edge domains. Table 1 provides a comprehensive summary of these representative multimodal models, detailing their core modalities, key strategies, and downstream capabilities.

In summary, multimodal feature embedding frameworks have progressively moved beyond theoretical concepts and benchmark evaluations to become practical engines guiding real biological discovery. By integrating evolutionary sequence information, three-dimensional structural features, chemical reaction semantics, and textual knowledge, these models not only significantly improve the accuracy of protein function annotation but also, more importantly, provide verifiable computational tools for elucidating the functions of uncharacterized proteins, rationally designing enzyme catalysts, and optimizing protein engineering. Emerging evidence suggests that this closed loop is already taking shape in practice. In the CAFA community challenge, computational predictions of uncharacterized protein functions have directly driven targeted experimental screens, resulting in new functional annotations for over 1000 genes, and the experimentally validated annotations accumulated in each round subsequently serve as refined benchmarks to improve the next generation of predictors [62]. More recently, ESM3 demonstrated a compelling instance of this paradigm at the molecular design level: by jointly reasoning over protein sequence, structure, and function tokens within a unified multimodal generative framework, the model was prompted to generate a novel green fluorescent protein esmGFP, whose sequence diverges from any known natural GFP by an estimated 500 million years of evolutionary distance, and whose fluorescent activity was subsequently confirmed through direct laboratory synthesis and characterization [63]. As multimodal technologies deepen their integration and the “AI prediction–experimental validation” closed loop continues to be refined, this paradigm is poised to play an increasingly central role in synthetic biology, drug discovery, and enzyme engineering.

**Table 1:** Summary of Multimodal Models.

No.	Model Name	Year	Core Modalities	Key Strategy/Architecture	Main Downstream Tasks
1	CLEAN [23]	2023	Seq	Supervised contrastive learning (Hard Negatives)	EC Prediction, Multifunctional Enzyme ID
2	ProTrek [24]	2024	Seq + Struct + Text	Tri-modal contrastive learning (Alignment)	Zero-Shot Retrieval, Function Prediction
3	GPSFun [37]	2024	Seq + Struct	Geometric graph network based on predicted structures	GO Prediction, Subcellular Localization
4	TransFun [40]	2023	Seq + Struct	SE(3)-equivariant GNN (Rotation Invariant)	EC/GO Prediction
5	LM-GVP [41]	2022	Seq + Struct	Geometric Vector Perceptron (GVP) structure head	Protein Property, GO Prediction
6	SaProt [42]	2024	Seq + Struct	Discretization of 3D structure into linguistic tokens	Stability, EC/GO Prediction
7	Prot2Text [57]	2023	Seq + Struct + Text	Encoder–Decoder generative architecture	Function Description Generation
8	ESM-GearNet [38]	2024	Seq + Struct	Geometric GNN guided by sequence semantics	EC/GO Prediction
9	ESM-AA [64]	2024	Seq + Struct	Atomic-level multi-scale joint pre-training	Affinity Prediction, Classification
10	EZSpecificity [65]	2025	Seq + 3D Complex	Cross-attention b/w sequence and binding pocket geometry	Substrate Specificity Prediction
11	DMMAFS [66]	2025	Seq + Struct	Dynamic multi-attention feature fusion network	GO Term Prediction
12	STELLA [67]	2025	Seq + Struct + Text	Multimodal instruction tuning (Text-Align)	Function Description Generation
13	ProteinGPT [68]	2024	Seq + Struct + Text	Large Multimodal Model (LMM) inspired by VLMs	Multifunctional Understanding and Generation
14	CataPro [69]	2025	Seq + Chemical	Molecular fingerprints integration + Attention correction	*k*_cat_, *K*_m_ Prediction
15	UniKP [70]	2023	Seq + SMILES	Dual-modal representation + Ensemble ML	*k*_cat_*, K*_m_, *k*_cat_/*K*_m_ Prediction
16	ProteinF3S [71]	2024	Seq + Struct + Surf	Bidirectional fusion (Seq/Struct/Surface)	Enzyme Reaction Class, EC Prediction
17	FusionProt [72]	2025	Seq + Struct	Serial Fusion + Contrastive Pretraining	EC/GO Prediction
18	CLAIRE [73]	2025	Seq + Reaction	Contrastive learning for enzyme-reaction alignment	Reaction Type Class, EC Prediction
19	MMKcat [74]	2025	Seq + Struct + SMILES	Feature Fusion Transformer (Non-uniform masking)	Turnover Number (*k*_cat_) Prediction
20	OneProt [75]	2025	Seq + Struct + Text	ImageBind-style multimodal alignment	Multimodal Alignment and Representation
21	ProMEP [76]	2024	Seq + Struct	Self-Supervised Pretraining + Chamfer Distance	Mutation Effect Prediction, Engineering
22	CACLENS [77]	2025	Seq + Chem + Text	Multi-task contrastive learning + Gated fusion	Reaction Classification, Enzyme Screening

Seq: Amino acid sequence (1D). Struct: Protein 3D structure (coordinates, contacts, or predicted folds). Chemical/SMILES: Chemical structure of ligands, substrates, or cofactors. Text: Scientific literature, functional descriptions, or natural language instructions. Reaction: Enzyme reaction equations or reaction type information. Surf: Protein surface features. EC: Enzyme Commission number (Function classification). GO: Gene Ontology (Molecular Function, Biological Process, etc.). *k*_cat_*/K*_m_: Enzyme kinetic parameters (Catalytic constant/Michaelis constant).

## 4. Current Challenges and Future Perspectives: Towards Physics-Informed Intelligence

**Figure 5:**
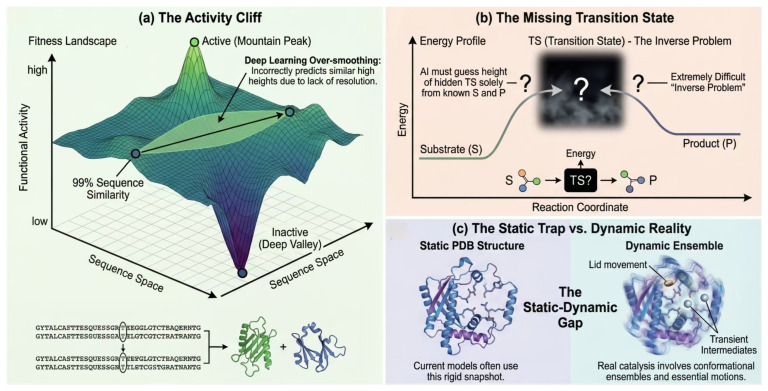
Fundamental Physical Barriers in Current Deep Learning Models. (**a**) The Activity Cliff: Conventional embeddings often smooth over the rugged fitness landscape, failing to distinguish functional variants from non-functional mutants despite high sequence similarity. (**b**) The “Inverse Problem” of Reaction Mechanisms: Models trained solely on ground-state substrates and products lack explicit supervision of the high-energy Transition State (TS), limiting their ability to predict catalytic rates (*k*_cat_). (**c**) The Static–Dynamic Gap: Relying on rigid crystal structures neglects the conformational ensembles and transient intermediate states (e.g., loop dynamics) essential for substrate binding and catalysis.

While multimodal embeddings have expanded the horizons of functional annotation, the field faces an “epistemic barrier”: current models excel at capturing statistical correlations but struggle with physical causality. As we push towards precision engineering, we must confront three fundamental bottlenecks: the ruggedness of fitness landscapes, the invisibility of transition states, and the neglect of conformational dynamics (Figure 5).

### 4.1. The “Activity Cliff” and Heterogeneous Alignment

In the realm of feature representation and spatial alignment, models confront fundamental challenges imposed by Activity Cliffs and the fusion of heterogeneous modalities. An Activity Cliff refers to the phenomenon within the protein fitness landscape where minimal structural perturbations—such as single-point mutations or atomic substitutions—trigger drastic, non-continuous changes in biological activity. This phenomenon directly challenges the “Similarity Principle” (the smoothness assumption) that underpins most deep learning architectures, serving as the primary reason for why high-homology inference frequently fails to distinguish functional variants.

Current mainstream PLMs (e.g., ESM-2, ProtTrans) tend to prioritize the capture of global folding patterns and evolutionary conservation. Consequently, the generated embeddings suffer from “over-smoothing” in the global structural space. This predominance of global features obscures the geometric subtleties of the local micro-environment that dictate enzyme–substrate specificity, rendering models incapable of differentiating between mutants with high sequence similarity but divergent functions [78]. Simultaneously, the heterogeneity of multimodal data comprising discrete sequence tokens, continuous geometric coordinates, and graph-structured reaction information resides in disparate feature spaces. Simple concatenation strategies are often insufficient to bridge this semantic gap. There is an urgent need to design high-order alignment strategies based on Contrastive Learning or to construct a Joint Embedding Space to achieve effective complementarity between these distinct physical modalities [59].

A further limitation concerns the chemical scope of the training corpus itself. Mainstream PLMs are pretrained almost exclusively on homochiral, canonical sequences, rendering their embeddings poorly calibrated, and their tokenizers structurally bind to heterochiral peptides containing D-residues, genetically encoded non-canonical amino acids (e.g., p-azido-phenylalanine used to anchor exogenous chromophores in artificial photoenzymes via click chemistry [79]), and macrocyclic or peptidomimetic scaffolds that deviate from standard backbone topology [80]. In such regimes, non-natural residues are typically collapsed onto their closest canonical neighbors, erasing precisely the chemical information that dictates the designed function. Addressing this boundary will require either tokenizer extensions coupled with targeted pretraining on chemically augmented corpora, or hybrid representations that concatenate PLM embeddings with explicit atom-level graphs of non-canonical moieties [81].

### 4.2. The Inverse Problem of Transition State Inference

To mitigate these deficiencies, recent studies have incorporated chemical reaction information (e.g., substrate/product molecular graphs) into prediction models [82]. While this has yielded marginal improvements, physically, it constitutes a formidable inverse problem. Models are forced to implicitly infer the properties of the ephemeral and highly unstable Transition State (TS) relying solely on the ground-state information of the reaction’s “start” (substrates) and “end” (products) [83]. Without explicit supervision regarding the energy barrier or the geometry of the transition state, the model’s ability to capture the true catalytic mechanism remains limited [84].

### 4.3. The Neglect of Conformational Dynamics

Furthermore, enzymes are not rigid entities but exist as dynamic Conformational Ensembles. Many enzymes execute their catalytic cycles through significant conformational rearrangements, such as domain rotation or “lid-opening/closing” mechanisms (Induced Fit). However, current Graph Neural Networks (GNNs) or Transformers predominantly rely on crystal structures (PDB) or AlphaFold-predicted structures as inputs. These structures represent “static snapshots” at thermodynamic equilibrium and fail to capture microscopic Conformational Dynamics [85]. This absence of dynamic information, compounded by the scarcity of high-quality kinetic data, prevents models from genuinely comprehending complex enzymatic properties such as allosteric regulation and temperature dependence [86].

### 4.4. Future Outlook: From Static Correlation to Dynamic Causality

In response to this impasse, frontier research is pivoting from “static correlations” toward Physics-Informed Machine Learning (PIML, modeling complex biological systems by combining parameterized physical laws with data-driven methods) and dynamic fusion. To ground models in physical reality, researchers are integrating thermodynamic constraints—such as energy conservation and microscopic reversibility—directly as regularization terms within loss functions. Additionally, energy parameters derived from transition state simulations are increasingly utilized as intermediate supervision signals to force the model to adhere to fundamental physicochemical laws [87]. Parallel to these constraint-based approaches, the integration of Molecular Dynamics (MD) simulations with AI has emerged as a robust trend for capturing conformational plasticity. By extracting “Dynamic Fingerprints” including Root Mean Square Fluctuation (RMSF) and pairwise distance distributions of key residues from short-term simulations, these hybrid architectures can explicitly model the flexibility of active sites, thereby overcoming the limitations of static structural inputs [88]. Ultimately, the next generation of enzyme function prediction must transcend the statistical correlation of sequences. By forging a symbiosis between the generative power of Language Models, the geometric intuition of Equivariant Networks, and the fundamental laws of Physical Chemistry, we are moving towards a ‘Digital Enzymologist’, an agent capable not just of annotation, but of reasoning about reaction mechanisms and designing catalysts de novo for a sustainable future. Notably, contemporary tools for de novo computational protein design increasingly rely on PLM and multimodal representation schemes, suggesting that function prediction and sequence generation are fundamentally two complementary readouts of a shared learned representation space [89]. Within this converging paradigm, multimodal function predictors can serve as active evaluation modules scoring the functional plausibility of designer sequences [63], while recent advances in automated protein design and in silico engineering provide essential methodological context for this intersection [90,91].

## 5. Conclusions

In summary, the transition from homology-based sequence alignment to multimodal deep learning represents a watershed moment in computational biology. As elucidated in this review, the traditional reliance on sequence identity is being supplanted by a holistic ecosystem that integrates PLMs with a diverse array of orthogonal modalities. By synergistically combining evolutionary semantics with geometric perception (to resolve structural subtleties), chemical reaction logic (to align enzyme–substrate spaces), and textual knowledge (to enable zero-shot reasoning), current architectures have successfully breached the “homology ceiling” that has long constrained the annotation of the dark proteome.

This integration has proven to be the definitive catalyst to accelerate the functional prediction of uncharacterized proteins. We are thus witnessing a fundamental paradigm shift: moving from retrieving known functions based on statistical similarity toward reasoning about novel functions grounded in physicochemical principles. Although challenges persist—particularly in bridging the “activity cliff” and closing the temporal gap between static structures and dynamic catalysis—the trajectory is unequivocal. The future of the field lies in evolving beyond “static classifiers” toward “dynamic, physics-informed engines,” capable not only of deciphering the biological mysteries of extant orphan proteins but also of engineering novel therapeutic candidates and biocatalytic tools that will drive the next generation of biotechnology and biomedicine.

## Data Availability

No new data were created or analyzed in this study. Data sharing is not applicable to this article.

## Abbreviations

The following abbreviations are used in this manuscript:

PLMs	Protein Language Models
PPIs	protein–protein interactions
GHs	Glycoside Hydrolases
pHMMs	profile hidden Markov models
ML	Machine Learning
DL	Deep Learning
NLP	Natural Language Processing
PSSM	Position-Specific Scoring Matrix
MSA	Multiple Sequence Alignment
MLMs	Masked Language Models
GNNs	Graph Neural Networks
GCNs	Graph Convolutional Networks
GO	Gene Ontology
EGNN	Equivariant Graph Neural Network
GVP	Geometric Vector Perceptron
SSNs	Sequence Similarity Networks
GBM	Gradient Boosting Model
DAG	Directed Acyclic Graph
KGs	Knowledge Graphs
TS	Transition State
PDB	Protein Data Bank
PIML	Physics-Informed Machine Learning
MD	Molecular Dynamics
RMSF	Root Mean Square Fluctuation

## References

[1] Ninck S., Klaus T., Kochetkova T.V., Esser S.P., Sewald L., Kaschani F., Bräsen C., Probst A.J., Kublanov I.V., Siebers B. (2024). Environmental activity-based protein profiling for function-driven enzyme discovery from natural communities. Environ. Microbiome.

[2] Yang J., Li F.-Z., Arnold F.H. (2024). Opportunities and challenges for machine learning-assisted enzyme engineering. ACS Cent. Sci..

[3] Bell E.L., Hutton A.E., Burke A.J., O’Connell A., Barry A., O’Reilly E., Green A.P. (2024). Strategies for designing biocatalysts with new functions. Chem. Soc. Rev..

[4] Katsimpouras C., Stephanopoulos G. (2021). Enzymes in biotechnology: Critical platform technologies for bioprocess development. Curr. Opin. Biotechnol..

[5] Nandi S., Bhaduri S., Das D., Ghosh P., Mandal M., Mitra P. (2024). Deciphering the lexicon of protein targets: A review on multifaceted drug discovery in the era of artificial intelligence. Mol. Pharm..

[6] Liu S., Rodriguez J.S., Munteanu V., Ronkowski C., Sharma N.K., Alser M., Andreace F., Blekhman R., Błaszczyk D., Chikhi R. (2025). Analysis of metagenomic data. Nat. Rev. Methods Primers.

[7] Drula E., Garron M.-L., Dogan S., Lombard V., Henrissat B., Terrapon N. (2022). The carbohydrate-active enzyme database: Functions and literature. Nucleic Acids Res..

[8] Wu C.H. (2006). The Universal Protein Resource (UniProt): An expanding universe of protein information. Nucleic Acids Res..

[9] Schneider M., Lane L., Boutet E., Lieberherr D., Tognolli M., Bougueleret L., Bairoch A. (2009). The UniProtKB/Swiss-Prot knowledgebase and its Plant Proteome Annotation Program. J. Proteom..

[10] Camacho C., Coulouris G., Avagyan V., Ma N., Papadopoulos J., Bealer K., Madden T.L. (2009). BLAST+: Architecture and applications. BMC Bioinform..

[11] Grewal J.K., Krzywinski M., Altman N. (2020). Markov models—Training and evaluation of hidden Markov models. Nat. Methods.

[12] Hamamsy T., Morton J.T., Blackwell R., Berenberg D., Carriero N., Gligorijevic V., Strauss C.E., Leman J.K., Cho K., Bonneau R. (2024). Protein remote homology detection and structural alignment using deep learning. Nat. Biotechnol..

[13] Neu D.A., Lahann J., Fettke P. (2022). A systematic literature review on state-of-the-art deep learning methods for process prediction. Artif. Intell. Rev..

[14] Chen J., Gu Z., Lai L., Pei J. (2023). In silico protein function prediction: The rise of machine learning-based approaches. Med. Rev..

[15] Altschul S.F., Madden T.L., Schäffer A.A., Zhang J., Zhang Z., Miller W., Lipman D.J. (1997). Gapped BLAST and PSI-BLAST: A new generation of protein database search programs. Nucleic Acids Res..

[16] Jumper J., Evans R., Pritzel A., Green T., Figurnov M., Ronneberger O., Tunyasuvunakool K., Bates R., Žídek A., Potapenko A. (2021). Highly accurate protein structure prediction with AlphaFold. Nature.

[17] Lin Z., Akin H., Rao R., Hie B., Zhu Z., Lu W., Smetanin N., Verkuil R., Kabeli O., Shmueli Y. (2023). Evolutionary-scale prediction of atomic-level protein structure with a language model. Science.

[18] Kulmanov M., Khan M.A., Hoehndorf R., Wren J. (2018). DeepGO: Predicting protein functions from sequence and interactions using a deep ontology-aware classifier. Bioinformatics.

[19] Cerri R., Barros R.C., de Carvalho A.C. Hierarchical multi-label classification for protein function prediction A local approach based on neural networks. Proceedings of the 2011 11th International Conference on Intelligent Systems Design and Applications.

[20] Nakano F.K., Lietaert M., Vens C. (2019). Machine learning for discovering missing or wrong protein function annotations. BMC Bioinform..

[21] Rives A., Meier J., Sercu T., Goyal S., Lin Z., Liu J., Guo D., Ott M., Zitnick C.L., Ma J. (2021). Biological structure and function emerge from scaling unsupervised learning to 250 million protein sequences. Proc. Natl. Acad. Sci. USA.

[22] Elnaggar A., Heinzinger M., Dallago C., Rehawi G., Wang Y., Jones L., Gibbs T., Feher T., Angerer C., Steinegger M. (2021). Prottrans: Toward understanding the language of life through self-supervised learning. IEEE Trans. Pattern Anal. Mach. Intell..

[23] Yu T., Cui H., Li J.C., Luo Y., Jiang G., Zhao H. (2023). Enzyme function prediction using contrastive learning. Science.

[24] Su J., He Y., You S., Jiang S., Zhou X., Zhang X., Wang Y., Su X., Tolstoy I., Chang X. (2025). A trimodal protein language model enables advanced protein searches. Nat. Biotechnol..

[25] Ryu J.Y., Kim H.U., Lee S.Y. (2019). Deep learning enables high-quality and high-throughput prediction of enzyme commission numbers. Proc. Natl. Acad. Sci. USA.

[26] Llinares-Lopez F., Berthet Q., Blondel M., Teboul O., Vert J. (2022). Deep-learning language models help to improve protein sequence alignment. Nat. Methods.

[27] Devlin J., Chang M.W., Lee K., Toutanova K. (2019). BERT pre-training of deep bidirectional transformers for language understanding. arXiv.

[28] Ferruz N., Schmidt S., Höcker B. (2022). ProtGPT2 is a deep unsupervised language model for protein design. Nat. Commun..

[29] Cheung M.S., Chavez L.L., Onuchic J.N. (2004). The energy landscape for protein folding and possible connections to function. Polymer.

[30] Vig J., Madani A., Varshney L.R., Xiong C., Socher R., Rajani N.F. (2020). Bertology meets biology: Interpreting attention in protein language models. arXiv.

[31] Fang X., Wang F., Liu L., He J., Lin D., Xiang Y., Zhu K., Zhang X., Wu H., Li H. (2023). A method for multiple-sequence-alignment-free protein structure prediction using a protein language model. Nat. Mach. Intell..

[32] Alley E.C., Khimulya G., Biswas S., AlQuraishi M., Church G.M. (2019). Unified rational protein engineering with sequence-based deep representation learning. Nat. Methods.

[33] Ibtehaz N., Kagaya Y., Kihara D. (2023). Domain-PFP allows protein function prediction using function-aware domain embedding representations. Commun. Biol..

[34] Capela J., Zimmermann-Kogadeeva M., Dijk A.D.v., de Ridder D., Dias O., Rocha M. (2025). Comparative assessment of protein large language models for enzyme commission number prediction. BMC Bioinform..

[35] Mao Y., Xu W., Shun Y., Chai L., Xue L., Yang Y., Li M. (2025). A multimodal model for protein function prediction. Sci. Rep..

[36] Gligorijević V., Renfrew P.D., Kosciolek T., Leman J.K., Berenberg D., Vatanen T., Chandler C., Taylor B.C., Fisk I.M., Vlamakis H. (2021). Structure-based protein function prediction using graph convolutional networks. Nat. Commun..

[37] Yuan Q., Tian C., Song Y., Ou P., Zhu M., Zhao H., Yang Y. (2024). GPSFun: Geometry-aware protein sequence function predictions with language models. Nucleic Acids Res..

[38] Zhang Z., Wang C., Xu M., Chenthamarakshan V., Lozano A., Das P., Tang J. (2023). A systematic study of joint representation learning on protein sequences and structures. arXiv.

[39] Song Y., Yuan Q., Chen S., Zeng Y., Zhao H., Yang Y. (2024). Accurately predicting enzyme functions through geometric graph learning on ESMFold-predicted structures. Nat. Commun..

[40] Boadu F., Cao H., Cheng J. (2023). Combining protein sequences and structures with transformers and equivariant graph neural networks to predict protein function. Bioinformatics.

[41] Wang Z., Combs S.A., Brand R., Calvo M.R., Xu P., Price G., Golovach N., Salawu E.O., Wise C.J., Ponnapalli S.P. (2022). Lm-gvp: An extensible sequence and structure informed deep learning framework for protein property prediction. Sci. Rep..

[42] Su J., Han C., Zhou Y., Shan J., Zhou X., Yuan F. (2023). Saprot: Protein language modeling with structure-aware vocabulary. bioRxiv.

[43] Mikhael P.G., Chinn I., Barzilay R. (2024). Clipzyme: Reaction-conditioned virtual screening of enzymes. arXiv.

[44] Hua C., Zhong B., Luan S., Hong L., Wolf G., Precup D., Zheng S. (2024). Reactzyme: A benchmark for enzyme-reaction prediction. Adv. Neural Inf. Process. Syst..

[45] Du Z., Fu W., Guo X., Caragea D., Li Y. (2025). FusionESP: Improved Enzyme–Substrate Pair Prediction by Fusing Protein and Chemical Knowledge. J. Chem. Inf. Model..

[46] Campbell M.J. (2024). VIPER: A General Model for Prediction of Enzyme Substrates. bioRxiv.

[47] Qian W., Wang X., Huang Y., Kang Y., Pan P., Hsieh C.-Y., Hou T. (2024). Deep Learning-Driven Insights into Enzyme–Substrate Interaction Discovery. J. Chem. Inf. Model..

[48] Pate S.C., Wang E.H., Broadbelt L.J., Tyo K.E. (2025). RC-GNN: A predictive model of enzyme-reaction pairs. bioRxiv.

[49] Paton A.E., Boiko D.A., Perkins J.C., Cemalovic N.I., Reschützegger T., Gomes G., Narayan A.R. (2025). Connecting chemical and protein sequence space to predict biocatalytic reactions. Nature.

[50] Huo M., Guo H., Cheng X., Singh D., Rahmani H., Li S., Gerlof P., Ideker T., Grotjahn D.A., Villa E. (2024). Multi-modal large language model enables protein function prediction. bioRxiv.

[51] Gu Y., Tinn R., Cheng H., Lucas M., Usuyama N., Liu X., Naumann T., Gao J., Poon H. (2021). Domain-specific language model pretraining for biomedical natural language processing. ACM Trans. Comput. Healthc..

[52] Zhang N., Bi Z., Liang X., Cheng S., Hong H., Deng S., Lian J., Zhang Q., Chen H. (2022). Ontoprotein: Protein pretraining with gene ontology embedding. arXiv.

[53] Zhou H.-Y., Fu Y., Zhang Z., Cheng B., Yu Y. Protein representation learning via knowledge enhanced primary structure reasoning. Proceedings of the Eleventh International Conference on Learning Representations.

[54] Kulmanov M., Guzmán-Vega F.J., Roggli P.D., Lane L., Arold S.T., Hoehndorf R. (2023). Deepgo-se: Protein function prediction as approximate semantic entailment. bioRxiv.

[55] Shaw P., Gurram B., Belanger D., Gane A., Bileschi M.L., Colwell L.J., Toutanova K., Parikh A.P. (2024). ProtEx: A retrieval-augmented approach for protein function prediction. bioRxiv.

[56] Zhang Z., Lu J., Chenthamarakshan V., Lozano A., Das P., Tang J. (2024). Protir: Iterative refinement between retrievers and predictors for protein function annotation. arXiv.

[57] Abdine H., Chatzianastasis M., Bouyioukos C., Vazirgiannis M. Prot2text: Multimodal protein’s function generation with gnns and transformers. Proceedings of the AAAI Conference on Artificial Intelligence.

[58] Wang Z., Zhang Q., Ding K., Qin M., Zhuang X., Li X., Chen H. Instructprotein: Aligning human and protein language via knowledge instruction. Proceedings of the 62nd Annual Meeting of the Association for Computational Linguistics (Volume 1: Long Papers).

[59] Xu M., Yuan X., Miret S., Tang J. Protst: Multi-modality learning of protein sequences and biomedical texts. Proceedings of the International Conference on Machine Learning.

[60] Zhang Z., Xu M., Jamasb A., Chenthamarakshan V., Lozano A., Das P., Tang J. (2022). Protein representation learning by geometric structure pretraining. arXiv.

[61] Ayres G., Munsamy G., Heinzinger M., Ferruz N., Yang K., Bergman B., Lorenz P. (2025). Annotating the microbial dark matter with HiFi-NN. Iscience.

[62] Zhou N., Jiang Y., Bergquist T.R., Lee A.J., Kacsoh B.Z., Crocker A.W., Lewis K.A., Georghiou G., Nguyen H.N., Hamid M.N. (2019). The CAFA challenge reports improved protein function prediction and new functional annotations for hundreds of genes through experimental screens. Genome Biol..

[63] Hayes T., Rao R., Akin H., Sofroniew N.J., Oktay D., Lin Z., Verkuil R., Tran V.Q., Deaton J., Wiggert M. (2025). Simulating 500 million years of evolution with a language model. Science.

[64] Zheng K., Long S., Lu T., Yang J., Dai X., Zhang M., Nie Z., Ma W.-Y., Zhou H. (2024). ESM All-Atom Multi-scale Protein Language Model for Unified Molecular Modeling. arXiv.

[65] Cui H., Su Y., Dean T.J., Yu T., Zhang Z., Peng J., Shukla D., Zhao H. (2025). Enzyme specificity prediction using cross-attention graph neural networks. Nature.

[66] He L., Deng Z., Hu F., Wang Z., Zuo Y., Zhu L., Chen H., Pan X., Wei Z., Wang L. (2025). DMMAFS: Protein Function Prediction Based on Multi-Modal Multi-Attention Fusion Features. IEEE Trans. Comput. Biol. Bioinform..

[67] Xiao H., Lin W., Chen X., Wang H., Chen K., Li J., Sun Y., Dai S., Wu B., Ye Q. (2025). STELLA Leveraging Structural Representations to Enhance Protein Understanding with Multimodal LLMs. bioRxiv.

[68] Xiao Y., Sun E., Jin Y., Wang Q., Wang W. (2024). ProteinGPT Multimodal LLM for Protein Property Prediction and Structure Understanding. arXiv.

[69] Wang Z., Xie D., Wu D., Luo X., Wang S., Li Y., Yang Y., Li W., Zheng L. (2025). Robust enzyme discovery and engineering with deep learning using CataPro. Nat. Commun..

[70] Yu H., Deng H., He J., Keasling J.D., Luo X. (2023). UniKP: A unified framework for the prediction of enzyme kinetic parameters. Nat. Commun..

[71] Yuan M., Shen A., Ma Y., Du J., An B., Wang M. (2025). ProteinF3S: Boosting enzyme function prediction by fusing protein sequence, structure, and surface. Brief. Bioinform..

[72] Kalifa D., Singer U., Radinsky K. (2025). FusionProt: Fusing Sequence and Structural Information for Unified Protein Representation Learning. bioRxiv.

[73] Zeng Z., Guo J., Jin J., Luo X. (2025). CLAIRE: A contrastive learning-based predictor for EC number of chemical reactions. J. Cheminformatics.

[74] Sun X., Wang Y.G., Shen Y. (2025). A multimodal deep learning framework for enzyme turnover prediction with missing modality. Comput. Biol. Med..

[75] Hu L., Flöge K., Udayakumar S., Sommer J., Piraud M., Kesselheim S., Fortuin V., Günnemann S., van der Weg K.J., Gohlke H. (2025). OneProt: Towards multi-modal protein foundation models via latent space alignment of sequence, structure, binding sites and text encoders. PLoS Comput. Biol..

[76] Cheng P., Mao C., Tang J., Yang S., Cheng Y., Wang W., Gu Q., Han W., Chen H., Li S. (2024). Zero-shot prediction of mutation effects with multimodal deep representation learning guides protein engineering. Cell Res..

[77] Yi X., Tan Y., Lin H., Zhang G., Tian Y., Wu A. (2025). CACLENS: A Multitask Deep Learning System for Enzyme Discovery. Adv. Sci..

[78] Meier J., Rao R., Verkuil R., Liu J., Sercu T., Rives A. (2021). Language models enable zero-shot prediction of the effects of mutations on protein function. Adv. Neural Inf. Process. Syst..

[79] Hoffmann J.-E. (2020). Bifunctional non-canonical amino acids: Combining photo-crosslinking with click chemistry. Biomolecules.

[80] Yang Z., Tian Z., Jia Y., Zhang T., Zheng J., Wang H., Su Y., He J., Liu L., Lan Y. (2026). Cross-Chirality Generalization by Axial Vectors for Hetero-Chiral Protein-Peptide Interaction Design. arXiv.

[81] Tang H., Boomsma W. (2025). UNAAGI: Atom-Level Diffusion for Generating Non-Canonical Amino Acid Substitutions. arXiv.

[82] Kroll A., Engqvist M.K., Heckmann D., Lercher M.J. (2021). Deep learning allows genome-scale prediction of Michaelis constants from structural features. PLoS Biol..

[83] Schwaller P., Probst D., Vaucher A.C., Nair V.H., Kreutter D., Laino T., Reymond J.-L. (2021). Mapping the space of chemical reactions using attention-based neural networks. Nat. Mach. Intell..

[84] Duan C., Du Y., Jia H., Kulik H.J. (2023). Accurate transition state generation with an object-aware equivariant elementary reaction diffusion model. Nat. Comput. Sci..

[85] Akdel M., Pires D.E., Pardo E.P., Jänes J., Zalevsky A.O., Mészáros B., Bryant P., Good L.L., Laskowski R.A., Pozzati G. (2022). A structural biology community assessment of AlphaFold2 applications. Nat. Struct. Mol. Biol..

[86] Mazurenko S., Prokop Z., Damborsky J. (2019). Machine learning in enzyme engineering. ACS Catal..

[87] Karniadakis G.E., Kevrekidis I.G., Lu L., Perdikaris P., Wang S., Yang L. (2021). Physics-informed machine learning. Nat. Rev. Phys..

[88] Malbranke C., Bikard D., Cocco S., Monasson R., Tubiana J. (2023). Machine learning for evolutionary-based and physics-inspired protein design: Current and future synergies. Curr. Opin. Struct. Biol..

[89] Kyro G.W., Qiu T., Batista V.S. (2025). A model-centric review of deep learning for protein design. arXiv.

[90] Kumar A., Ranbhor R., Patel K., Ramakrishnan V., Durani S. (2017). Automated protein design: Landmarks and operational principles. Prog. Biophys. Mol. Biol..

[91] Ranbhor R., Venkatesan R., Redkar A.S., Ramakrishnan V. (2025). Computational protein design: Advancing biotechnology through in silico engineering. Prog. Biophys. Mol. Biol..

